# Assessing organ-level immunoreactivity in a rat model of sepsis using TSPO PET imaging

**DOI:** 10.3389/fimmu.2022.1010263

**Published:** 2022-11-10

**Authors:** Neysha Martinez-Orengo, Sarine Tahmazian, Jianhao Lai, Zeping Wang, Sanhita Sinharay, William Schreiber-Stainthorp, Falguni Basuli, Dragan Maric, William Reid, Swati Shah, Dima A. Hammoud

**Affiliations:** ^1^ Center for Infectious Disease Imaging, Radiology and Imaging Sciences, Clinical Center, National Institutes of Health, Bethesda, MD, United States; ^2^ Chemistry and Synthesis Center, National Heart, Lung, and Blood Institute, National Institutes of Health, Rockville, MD, United States; ^3^ Flow and Imaging Cytometry Core Facility, National Institute of Neurological Disorders and Stroke, National Institutes of Health, Bethesda, MD, United States

**Keywords:** sepsis, TSPO (18 kda translocator protein), 18F-DPA-714, whole body PET/CT, organ-level immunoreactivity

## Abstract

There is current need for new approaches to assess/measure organ-level immunoreactivity and ensuing dysfunction in systemic inflammatory response syndrome (SIRS) and sepsis, in order to protect or recover organ function. Using a rat model of systemic sterile inflammatory shock (intravenous LPS administration), we performed PET imaging with a translocator protein (TSPO) tracer, [^18^F]DPA-714, as a biomarker for reactive immunoreactive changes in the brain and peripheral organs. *In vivo* dynamic PET/CT scans showed increased [^18^F]DPA-714 binding in the brain, lungs, liver and bone marrow, 4 hours after LPS injection. Post-LPS mean standard uptake values (SUV_mean)_ at equilibrium were significantly higher in those organs compared to baseline. Changes in spleen [^18^F]DPA-714 binding were variable but generally decreased after LPS. SUV_mean_ values in all organs, except the spleen, positively correlated with several serum cytokines/chemokines. *In vitro* measures of TSPO expression and immunofluorescent staining validated the imaging results. Noninvasive molecular imaging with [^18^F]DPA-714 PET in a rat model of systemic sterile inflammatory shock, along with *in vitro* measures of TSPO expression, showed brain, liver and lung inflammation, spleen monocytic efflux/lymphocytic activation and suggested increased bone marrow hematopoiesis. TSPO PET imaging can potentially be used to quantify SIRS and sepsis-associated organ-level immunoreactivity and assess the effectiveness of therapeutic and preventative approaches for associated organ failures, *in vivo*.

## Introduction

Sepsis is an abnormal response by the host immune system to microbial infections which frequently results in multi-organ dysfunction and death. As reported by the CDC, 1.7 million adults develop sepsis and more than 200,000 die in the USA every year in addition to millions of adults and children around the world (1, 2). Globally, sepsis is responsible for 20% of all-cause deaths and since 2017 it has been recognized by the World Health Organization as a global health concern (3, 4). Despite the vast improvement in septic patient outcomes over the last few decades, physical and psychological post-sepsis symptoms persist and remain a major problem affecting the quality of life of recovered patients (5, 6). The exact etiologies underlying neurocognitive and other systems’ dysfunctions in this patient population, including more recently, the survivors of moderate to severe COVID-19 infection, have been difficult to pinpoint (7).

Although an inflammatory circulatory process is an established manifestation of systemic inflammatory response syndrome (SIRS) and sepsis, the degree of direct organ-level inflammation is not easily inferred until late in the disease process when irreversible organ failure is impending (8). In 2016, the society of Critical Care Medicine and the European Society of Intensive Care Medicine prioritized organ dysfunction in their new definition of sepsis (Sepsis-3) which uses a sequential organ failure assessment (SOFA) score as an index (9). This led to calls for a new direction of research where assessing organ level inflammation and ensuing dysfunction becomes a priority along with attempts to protect or recover organ function (10). In this context, imaging studies using pre-clinical models of sepsis and biomarkers of peripheral immunoreactivity can be used to detect and gauge organ-level inflammation and consequently the effectiveness of various therapeutic and preventative approaches for SIRS/sepsis *in vivo*.

The 18kDa translocator protein (TSPO), formerly known as peripheral benzodiazepine receptor (PBR), is an outer mitochondrial membrane receptor expressed in many cell types, but especially known to be expressed in brain microglia (11, 12). Due to increased expression in activated microglia, TSPO is widely used as a PET imaging target in the detection and *in vivo* quantification of neuroinflammation in a variety of neuropathologies, and as therapeutics (13–20). However, as we have recently shown, TSPO is expressed not just in microglia, monocytes, and macrophages but also in dendritic cells, neutrophils, B- and T-cells, both in humans and macaques (21, 22). Extracranially, TSPO is also expressed in various cell types in the bone marrow (23) and other peripheral tissues (24) including heart (25), colon (26), liver (27), and lungs (28). Nevertheless, TSPO targeting in PET has been sparsely used to evaluate peripheral inflammation in preclinical and clinical models, with only a handful of published studies (26, 28–32), none of which focus on sepsis-induced inflammation in peripheral organs, beyond the lungs.

In this study, we evaluated the well-known TSPO PET ligand [^18^F]DPA-714 to quantify organ level immunoreactivity *in vivo*, after intravenous LPS injection. We corroborated our imaging findings with *ex-vivo* assessment of TSPO gene expression and correlated binding with various biomarkers of disease and inflammatory changes. Tissue sections of the brain, lung, and spleen were also assessed by immunofluorescence staining to explain our *in vivo* findings. Only one time point after LPS injection was used since our study is meant as a proof of concept.

## Methods

### Animals

Male Fisher rats were purchased from Charles River Laboratory (Wilmington, MA) and were housed in a temperature-controlled environment with free access to food and water with a 12-hour dark/light cycle. A total of 13 animals (Age range: 3.7-4.2 months, mean age: 3.96 ± 0.17 months; Weight range: 0.29-0.36 kg, mean weight 0.32 ± 0.01 kg) were used for all PET imaging experiments. An additional set of 15 animals (5 controls and 10 LPS-treated) were used to increase sample size for molecular experiments including cytokines/chemokines panels, qPCR, and immunohistochemistry (IHC).

### LPS administration

The LPS was extracted from E. coli serotype O111:B4 and purified by gel filtration (Sigma Aldrich #L3012). This serotype can stimulate B-cells and other cells of the immune system mainly *via* activation of Toll-like receptor 4 (TLR4), a receptor that recognizes Pathogen-associated molecular patterns (PAMPs).

The administration of either intraperitoneal (IP) or intravenous (IV) injection of LPS in rodents is commonly used to induce a systemic sterile inflammatory shock and organ failure that simulates sepsis. Although IP injections are easier and more convenient, IV injections allow for more consistent levels of LPS in the blood as well as faster induction of an immune response and neuroinflammation (33). Acute LPS injections in rats have shown to induce significant systemic and central inflammation, including different regions of the brain, as early as 2 hours after inoculation (34, 35). In human studies, the typical route of LPS injection is IV as well. In our study, we used the IV injection method in rats as described by others (36, 37), using 5mg/kg LPS dose. Since the main focus of our study was to evaluate organ level inflammation, LPS injection of rats *via* the IV route was deemed a suitable model to induce an immune response with increased cytokine levels in serum and tissues. In our hands, this resulted in all animals developing a measurable systemic and organ-level inflammatory syndrome within 4 hours.

### [^18^F]DPA-714 radioligand synthesis

[^18^F]DPA-714 was synthesized as previously reported (38). In a typical reaction, starting with 9600 MBq of fluorine-18, we obtained 3100 MBq of the product with a radiochemical purity > 99%. The molar activity was 125000 MBq/µmol.

### [^18^F]DPA-714 PET imaging

Prior to each scan, the animal was anesthetized (3-4% isoflurane) and the lateral tail vein was cannulated with a butterfly catheter connected to a heparin lock. Once the animal was properly positioned, [^18^F]DPA-714 was injected slowly through the tail vein catheter (mean dose 1.13 ± 0.1 mCi) over a period of 30 seconds as a bolus followed by a quick saline flush (300 µL). PET imaging using Inveon PET/CT scanner (Siemens Medical Solutions, USA) with a transaxial and axial field of view (FOV) of 10 and 12.7 cm, full width at half maximum spatial resolution at 1.4 mm center FOV, was initiated immediately after the injection. Dynamic PET scans were performed for 60 minutes.

Baseline [^18^F]DPA-714 scans were obtained for each animal. Two days following the baseline scan, animals received a prophylactic subcutaneous injection of buprenorphine (0.1 mg/kg) one hour before LPS was administered *via* intravenous tail vein (5mg/kg). After a 4-hour waiting period, [18F]DPA-714 PET/CT imaging was performed. Whole blood was also collected before and 4 hours after LPS injections. After completion of the baseline PET imaging session, the animals were allowed to recover whereas following the post-LPS scans, the animals were immediately euthanized and perfused with saline for whole blood and organ collection. Following this, various organs were collected and immediately snap frozen in liquid nitrogen. The tissues were stored at -80°C until further use for downstream procedures such as RNA extraction and lysate preparation. A separate group of animals (control n=4; LPS n=4) were used for immunohistochemistry. The detailed procedures for organ collection and tissue treatments prior to staining are mentioned below under “multiplex fluorescence immunohistochemistry”.

### [^18^F]DPA-714 PET image analysis

The images were reconstructed using OSEM-3D and were analyzed using PMOD 3.8 (PMOD Technologies, Ltd., Zurich, Switzerland). The PET images were co-registered to the CT image, and volumes of interest (VOIs) were drawn for the whole brain, liver, lungs, spleen, and bone marrow. Time activity curves (TACs) were derived from the dynamic images for each VOI. The mean standardized uptake at equilibrium was averaged from 26-40 min and reported as the SUV_mean_.

It is known that TSPO is expressed in the kidneys and can be upregulated in inflammatory conditions. These changes can usually be quantified by PET when kidney function is otherwise intact. In our study, however, we did not assess kidney binding, mainly because many of our animals showed decreased kidney function after LPS administration, a commonly seen phenomenon in SIRS and sepsis patients (sepsis-induced acute kidney injury (AKI)) (39). As a result of the secondary reduced glomerular filtration rate and tubular dysfunction in the kidneys, the effective excretion of our ligand and its metabolites was delayed, resulting in ligand retention in the parenchyma of the kidneys, as has been described with 99mTc-Mag3 scans (40). We thus assumed that the increased radioactivity in the kidneys four hours post-LPS likely reflects a combination of upregulated TSPO expression (due to inflammatory changes) and ligand retention within the renal parenchyma, and as such is unreliable as a measure of immune activation after LPS administration.

### Serum and lysate preparation

Serum from whole blood, as well as liver, brain, spleen, and lung lysates were collected from 13 LPS injected rats and 5 control rats. Pre- and post-LPS inoculation serum was collected for 8 animals and only post-LPS serum was collected for 10 animals. Sectional tissues with a total weight of 50 mg were obtained from each organ (lung, liver, spleen, and basal ganglia of the brain) and homogenized to perform RNA and protein extraction. Total cellular RNA from LPS treated rats and controls were isolated using the Zymo ZR-Duet DNA/RNA MiniPrep Plus Kit (Catalog No. D7003) according to the manufacturer’s instructions. The RNase-Free DNase Set (Qiagen No.79254) was used to remove genomic DNA from the RNA samples. The protein lysates were obtained by homogenizing the tissues in a protein extraction buffer and then collecting the supernatants after centrifugation. Total protein concentrations of the lysates were measured using the BCA assay (Pierce cat#23225) prior to performing enzyme-linked immunoassay (ELISA).

### Enzyme-linked immunoassay

Cytokine/chemokine levels were measured in the brain, liver, spleen, lung lysates, and in the serum of LPS treated and control rats. A multiplex ELISA kit (Millipore Sigma #RECYMAG65K27PMX) for 27 analytes was used following the manufacturer’s instructions. The panel included G-CSF, Eotaxin, GM-CSF, IL-1α, Leptin, MIP-1α, IL-4, IL-1B, IL-2, IL-6, EGF, IL-13, IL-10, IL-12p70, IFNγ, IL-5, IL-17, IL-18, MCP-1, IP-10, GRO/KC, VEGF, Fractalkine, LIX, MIP-2, TNFα, and RANTES analytes. The protein concentrations for the organ lysates were adjusted to 2mg/ml before analysis by ELISA. The serum samples were not diluted before the run. The plates were read on Bioplex 200™ (Bio-Rad) and the analyte concentrations were determined for all the organs and serum.

### Quantitative polymerase chain reaction of organ tissues

Synthesis of first-strand cDNA from total RNA was performed using RT^2^ First Strand Kit and the cDNA was amplified with RT² SYBR^®^Green qPCR Mastermix (Qiagen, Hilden, Germany). The housekeeping gene for the ribosomal protein lateral stalk subunit P1, *Rplp1* (Qiagen #PPR42363C-200) was used as an internal control. Samples for the gene of interest, *Tspo* (Qiagen #PPR06787A-200), were run in triplicates. Using CFX96 Real-time qPCR System (Bio-Rad, Hercules, CA), relative changes in mRNA expression levels were quantified. The Ct values were normalized to the housekeeping gene.

### Multiplex fluorescence immunohistochemistry

Animals were perfused with saline followed by 4% PFA. Tissues were cryoprotected using 10-30% sucrose gradient before being embedded in optimal cutting temperature compound (OCT), frozen, and cut in 10 μm-thick sections. Tissue slices from the brain, spleen, and lungs of a group of LPS injected animals (n=4) and control animals (n=3) were stained using different combinations of up to 5 primary antibodies to detect specific immunoinflammatory cell types using MF-IHC. These antibodies respectively included CD3 for T-cells (Thermo Fisher Scientific # MA1-7630), B220 for B-cells (Thermo Fisher Scientific # 14-0460-82), granulocyte marker for neutrophils (Thermo Fisher Scientific # 14-0570-82), CD68 for monocytes/macrophages (Abcam # ab125212), MHCII for dendritic cells (Thermo Fisher Scientific # 14-0920-82), and Iba1 for microglia (Cedarlane Labs # 234006(SY)). TSPO antibody (Abcam #ab109497) was used to stain for the protein. Each of the above primary immunoreactions was visualized using appropriate fluorophore-conjugated secondary antibodies obtained either from Jackson ImmunoResearch (DyLight 405 # 115-475-075) or Thermo Fisher Scientific/Invitrogen (Alexa Fluor 546 # A21123, Alexa Fluor 594 # A21145, Alexa Fluor 488 # A21151, Alexa Fluor 430 # A11064, Alexa Fluor 555 # A21435, Pacific Orange # P31584) and all antibodies were diluted based on the manufacturer’s recommendation. The cell nuclei were counterstained using 1 μg/ml DAPI to facilitate cell counting. All fluorescence signals were imaged using an Axio Imager.Z2 upright scanning wide field fluorescence microscope (Zeiss) equipped with Orca Flash 4.0 high resolution sCMOS camera (Hamamatsu), 200W X-cite 200DC broadband light source (Lumen Dynamics) and standard DAPI, and various Alexa Fluor filter sets (Semrock). After imaging, the multichannel image datasets were processed for image stitching, illumination correction, and the images were imported into Adobe Photoshop CS6 to produce pseudo-colored multi-channel composites.

### Staining quantification

Quantification of percent fluorescence intensities was performed using NIH ImageJ 1.53a software. For the lungs, a single ROI encompassing the whole tissue region was drawn. For the spleen, the white pulp and red pulp were analyzed with four ROIs drawn on each region and then combined. In the brain, ROIs were also drawn by region, including either the striatum, cortex, or corpus callosum; with multiple small ROIs drawn in each region. Liver IHC could not be performed due to high levels of autofluorescence prohibiting meaningful staining of different cell markers.

ImageJ was also used to quantify microglial length using the free hand lines tool to measure 80 ramifications per animal and the free hand selections tool to delineate 20 somas per animal from different ROIs of brain tissues.

### Statistics

Paired t-test was used to evaluate the differences in average [^18^F]DPA-714 binding (SUV_mean_) at baseline and post LPS administration for each organ. Unpaired t-test was used to compare TSPO mRNA expression between controls and LPS groups and to analyze the differences in TSPO and various cell marker stains by IHC. For those tests, p-values <0.05 were considered statistically significant. Even though we found colocalization of TSPO with different cell markers, we felt that our histopathology sample size was too small (controls n=3, LPS n=4) for an accurate and reliable Pearson analysis of TSPO binding.

Non-parametric Mann Whitney test was used to compare cytokine expression in serum and organ lysates between controls and LPS groups since many datasets were not normally distributed. p-values <0.01 were considered statistically significant.

Repeated measures correlations between SUV values and serum cytokine levels were performed using the *rmcorr* program in R (version 3.5.1). In order to account for multiple comparisons in this analysis, correlations with p-values < 0.01 were considered to reflect positive or negative associations.

### Study approval

All procedures were approved by the Animal Care and Use Committee (ACUC) of the Clinical Center (CC) at the National Institutes of Health (NIH) and were performed in an AAALAC International accredited facility in accordance with relevant NIH policies and the Animal Welfare Act and Regulations.

## Results

### Assessment of whole body TSPO distribution by [^18^F]DPA-714 PET imaging

The animals underwent PET/CT scans at baseline and 4 hours post-LPS injection. Post-LPS inoculation, the rats displayed reduced physical activity and slower respiratory rate (avg 30-35 breaths per minute) during the scan compared to baseline scans (breath rate 40-50 breaths per minute) under similar levels of anesthesia (1.5- 2% isoflurane-O_2_ mixture). Some of the animals also had diarrhea after LPS treatment.

**Figure 1:**
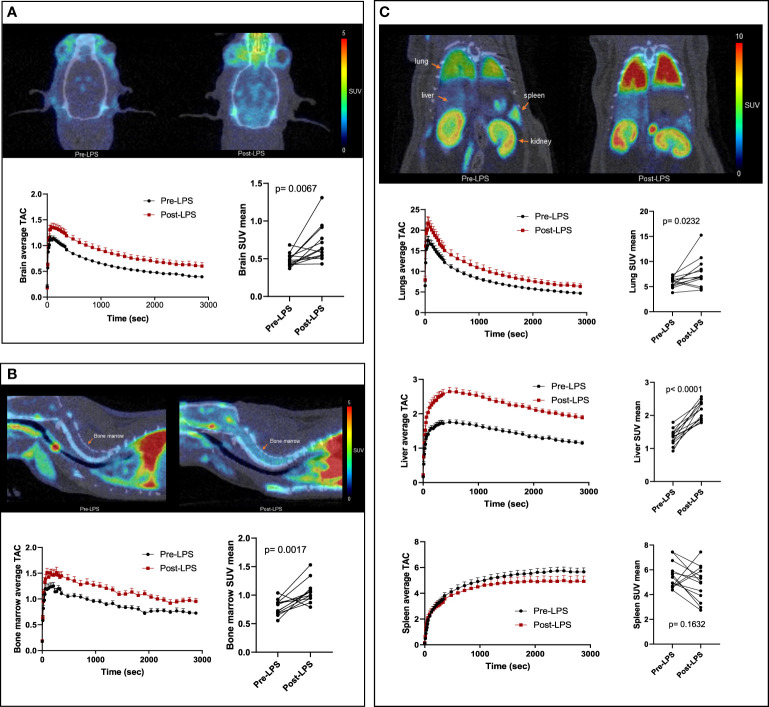
[^18^F]DPA-714 binding in the brain and peripheral organs of LPS-treated rats. Representative PET/CT scans (top), average time activity curves (TACs) (bottom left) and mean standardized uptake values (SUVs) of [^18^F]DPA-714 averaged from 26-40 minutes (bottom right) are shown in the brain **(A)**, bone marrow **(B)**, and peripheral organs- lungs, liver and spleen **(C)**. Representative PET images and mean TACs show increased binding in brain, lungs, liver, and bone marrow, but not in spleen, compared to baseline (n=13). Statistical analysis was performed using paired t-test to evaluate the differences in average [^18^F]DPA-714 binding by PET at baseline and post-LPS for each organ. p-values <0.05 are considered statistically significant. *p<0.05, **p<0.01, ****p<0.0001.

The post-LPS scans showed increased [^18^F]DPA-714 binding in the brain, lungs, liver, and bone marrow as demonstrated in the time activity curves (TACs) (**Figure 1**). In the spleen, most animals (10 out of 13) showed decreased binding rather than increased binding. On average, binding was decreased compared to baseline on the mean TAC. Mean TACs for whole brain, liver, lungs, and bone marrow showed higher [^18^F]DPA-714 binding in the post-LPS rats when compared to baseline. Post-LPS SUV_mean_ were significantly higher for brain (p =0.007), lungs (p=0.023), liver (p<0.0001), and bone marrow (p=0.002) with an average of 2-3-fold increase. There were no significant differences in spleen SUV_mean_ values with most animals instead showing decreased SUV_mean_ compared to baseline (**Figures 1A-C**).

### Changes in blood cell counts and cytokine levels reflect systemic inflammation

In our animals, there were significant decreases in platelets, white blood cells counts as well as monocytes, eosinophils and lymphocyte counts (**Supplementary Figure 1**).

**Figure 2:**
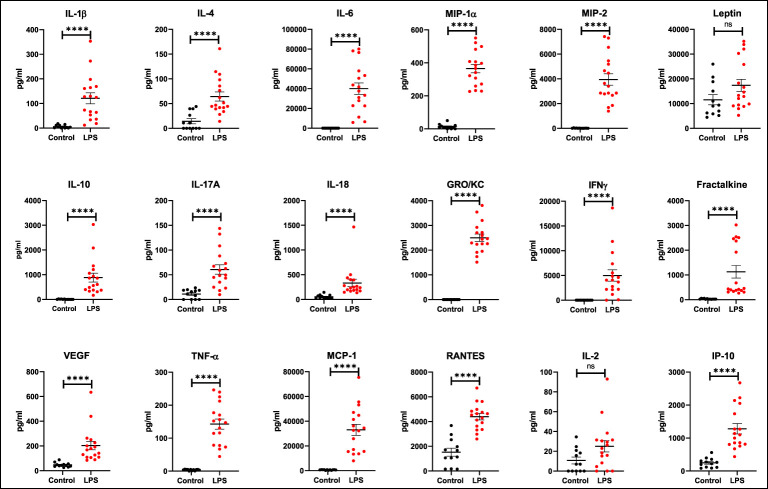
Expression of serum cytokines and chemokines. Increased expression (pg/mL) of various cytokines and chemokines is seen after LPS treatment (Control n=12, LPS n=17). Statistical analysis was performed using unpaired Mann Whitney test to evaluate changes in serum cytokines and chemokines between controls and LPS groups. Mean with SEM are shown. p-values <0.01 are considered statistically significant. ****p<0.0001, not significant (ns).

**Figure 3:**
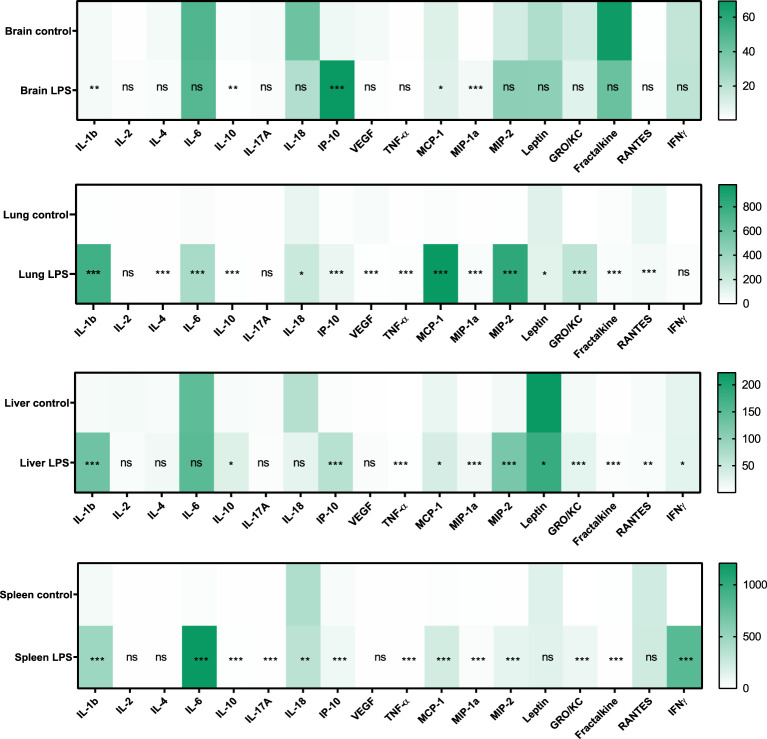
Expression of cytokines and chemokines in organ lysates. Changes in the expression (pg/mL) of various cytokines and chemokines in brain, lung, liver, and spleen lysates due to LPS treatment is shown (Control n=4, LPS n=14). Statistical analysis was performed using Mann-Whitney test to evaluate changes in tissue cytokines and chemokines between controls and LPS groups. p-values <0.01 are considered statistically significant. *p<0.05, **p<0.01, ***p<0.001, not significant (ns).

It has been demonstrated that both pro- and anti-inflammatory cytokines play an important role during sepsis and that serum levels increase in patients with sepsis. In our model, serum cytokine levels increased in the LPS group when compared to controls (**Figure 2**). The following list of serum cytokines showed significantly increased expression after LPS exposure: IL-1β, IL-4, IL-6, MIP-1α, MIP-2, IL-10, IL-17A, IL-18, GRO/KC, IFNγ, Fractalkine, VEGF, TNFα, MCP-1, RANTES, and IP-10 (all p<0.0001). Rats that did not have baseline measures also showed increased cytokine levels when compared to controls. At the organ level, increased expression of various cytokines in organ lysates from brain, lungs, liver, and spleen was also observed in the LPS group when compared to controls (**Figure 3**).

**Figure 4:**
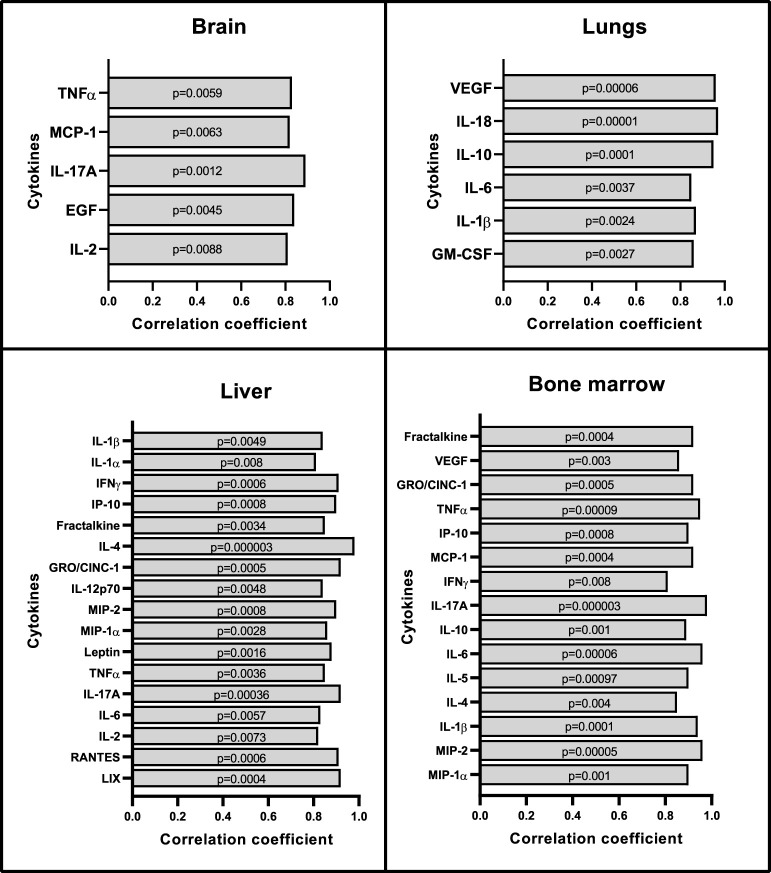
Correlation between organ SUV_mean_ with serum cytokines and chemokines. Repeated measures correlations analysis shows positive correlations between cytokines in serum and SUV_mean_ values from brain, lungs, liver, and bone marrow (n=8). No correlations were seen with the spleen. Statistical analysis was performed using the rmcorr package in R. In order to account for multiple comparisons in this analysis, correlations with p-values < 0.01 were considered to reflect positive or negative association.

Multiple serum cytokine levels positively correlated with SUV_mean_ of the brain, lungs, liver, and bone marrow (**Figure 4**). Some of the most relevant and frequently expressed cytokines that showed significant associations include IL-2, IL-17A, TNFα, IL-6, IL-1β, MCP-1, and IL-4. These cytokines have been associated with severity of sepsis, organ dysfunction, and mortality in septic shock patients (41). Additional serum cytokines that correlated with SUVs in specific organs included EGF (brain); GM-CSF, IL-10, IL-18, VEGF (lungs); IL-1α, Leptin, MIP-1α, IL-12p70, IFNγ, IP-10, GRO/KC, Fractalkine, LIX, MIP-2, and RANTES (liver); and MIP-1α, MIP-2, IL-5, IL-10, IFNγ, IP-10, GRO/KC, VEGF, and Fractalkine (bone marrow). No correlations were observed between cytokine levels in serum and spleen SUV_mean_ values.

### *Ex-vivo* assessment of organ level changes in TSPO expression

**Figure 5:**
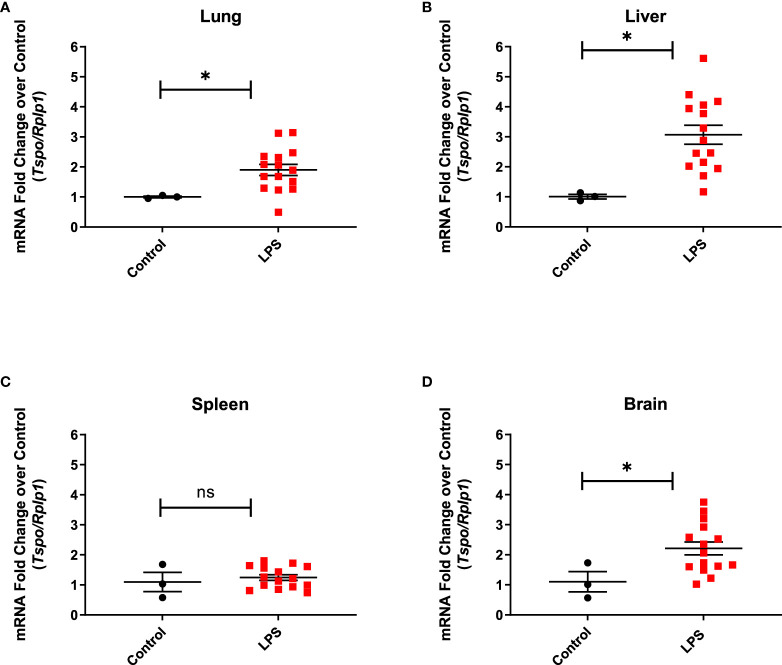
TSPO gene expression in organ lysates. Increased TSPO mRNA levels in the lung. **(A)** (p=0.0497), liver **(B)** (p=0.0121), and brain **(D)** (0.0462) from LPS treated rats (n=15), but not in the spleen **(C)** (p=0.5529), when compared to controls (n=3). Statistical analysis was performed using unpaired t-test. Mean with SEM are shown. p-values <0.05 are considered statistically significant. Tspo is target gene and ribosomal protein lateral stalk subunit P1 (Rplp1) is the housekeeping gene used for normalization. *p<0.05; not significant (ns).

Real time PCR performed to assess the changes in TSPO expression at the transcriptional level showed one to two-fold upregulation of TSPO mRNA expression in the lung (p=0.0497), liver (p=0.0121), and brain (p=0.0462) when compared to controls, but not in the spleen (p=0.5529), which is consistent with the PET imaging results (**Figure 5**).

**Figure 6:**
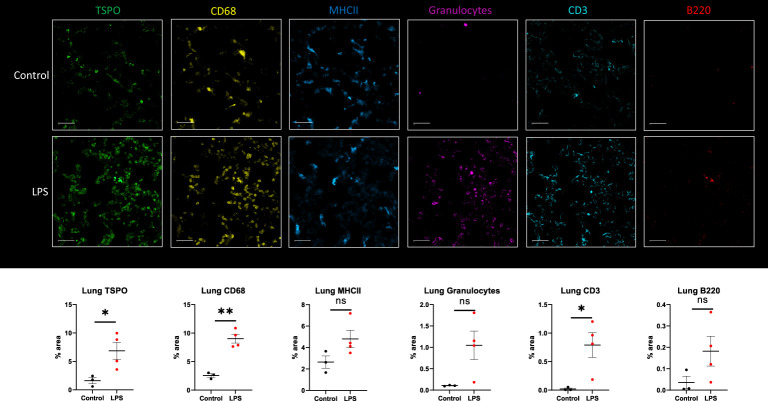
Immunohistochemistry in lung tissue sections. Representative MF-IHC images of a single lung section from control (top panels) and LPS treated rat (bottom panels). Quantification of the various stains was obtained by measuring whole lung section using Image J. Percentage area values are shown under each panel for TSPO, CD68 (macrophages), MHCII (dendritic cells), Granulocytes (neutrophils), CD3 (T cells), and B220 (B cells). There is increased TSPO (p=0.0361), CD68-monocytes/macrophages (p=0.001), and CD3-T cells (p=0.0305) staining in the lungs of LPS-treated rats (n=4) compared to controls (n=3). In this specific animal, staining for TSPO, macrophages, MHCII, granulocytes and lymphocytes was increased. Statistical analysis was performed using unpaired t-test. Mean with SEM are shown. p-values <0.05 are considered statistically significant. *p<0.05, **p<0.01, not significant (ns.)

Based on immunohistochemistry, there was significantly increased expression of TSPO (p=0.0361), CD68 (macrophage marker) (p=0.001) and CD3 (T cell marker) (p=0.0305) in the lungs of LPS treated animals when compared to controls (**Figure 6**). Additionally, the expression of B cells -B220 (p=0.1510), neutrophils -granulocytes (p=0.0626), and dendritic cells -MHCII (p=0.1018) was also higher compared to controls, although the differences did not reach statistical significance (**Figure 6**). While co-localization of TSPO staining with macrophages was the most noticeable, we also found co-localization of TSPO staining with dendritic cells, neutrophils (**Supplementary Figure 2**) and lymphocytes (**Supplementary Figure 3**).

**Figure 7:**
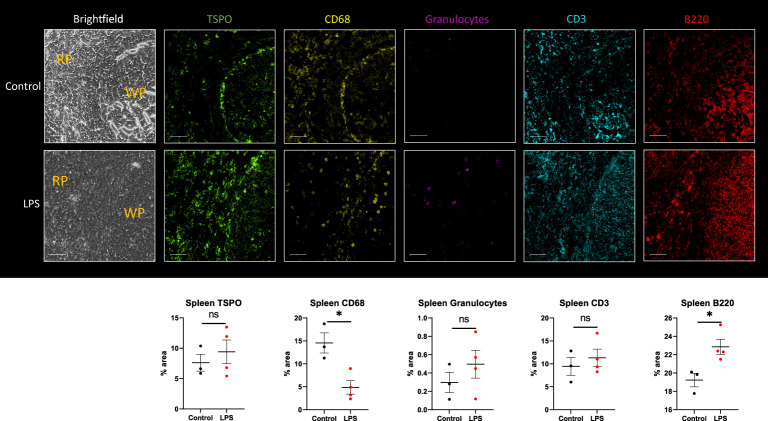
Immunohistochemistry in spleen tissue sections. Representative MF-IHC images of a single spleen section from control (top panels) and LPS-treated rat (bottom panels). The regions shown here include both the red pulp (RP) and white pulp (WP) areas as indicated in the brightfield images. Quantification of the various stains was obtained by measuring multiple ROIs in the red and white pulp spleen section using Image J. Percentage area values are shown under each panel for TSPO, CD68 (macrophages), MHCII (dendritic cells), Granulocytes (neutrophils), CD3 (T cells), and B220 (B-cells). No significant statistical difference in TSPO expression between groups is seen with two out of four animals showing increased staining, as shown. This is seen despite significant decreased immunoreactivity for CD68-monocytes/macrophages (p=0.0003), likely due to increased lymphocytic expression in the LPS group (n=4) compared to controls (n=3). Statistical analysis was performed using unpaired t-test. Mean with SEM are shown. p-values <0.05 are considered statistically significant. *p<0.05, not significant (ns).

We also analyzed the spleen by IHC using combined ROIs equally distributed between the red pulp and white pulp. There was decreased expression of monocytes/macrophages -CD68 (p=0.0121) and increased expression of B cells -B220 (p=0.0256) (**Figure 7**). Due to mixed response patterns, there were no statistically significant differences in the expression of TSPO, neutrophils -granulocytes, or T cells -CD3 in the spleen of LPS rats when compared to controls (**Figure 7**). Increased proliferating B cells in the lymphoid white pulp most likely represents the initiation of immune responses. This increase could have offset the loss of monocytes, resulting in no appreciable change in total TSPO expression in two out of four animals. The other two animals showed increased TSPO staining despite decreased CD68 staining. At the same time, they showed increased granulocyte and lymphocyte staining, possibly offsetting the decreased monocyte staining (**Figure 7**).

**Figure 8:**
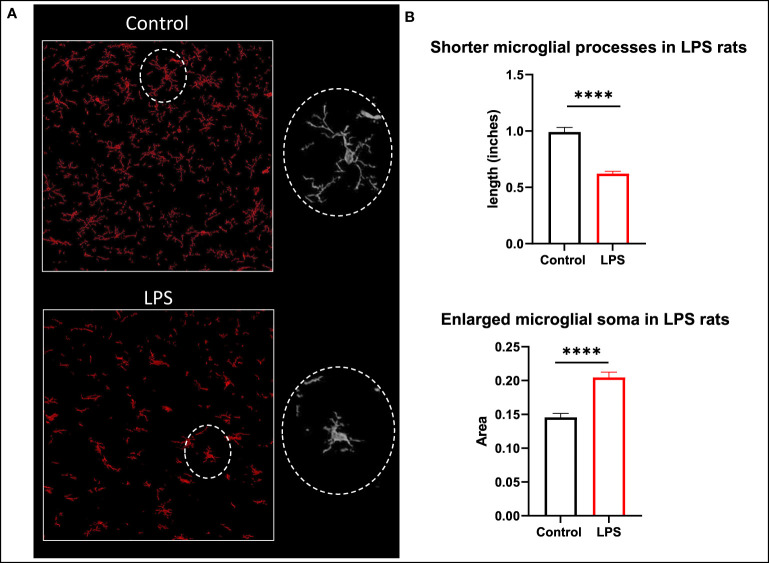
Morphological changes in microglia. **(A)** Representative images of microglia (Iba1 in red) in the corpus callosum of control and LPS treated rats. **(B)** Animals with systemic inflammation (LPS) show shorter microglial processes and thicker cell body compared to controls. Quantification was performed using the free hand selection tool from Image J to measure the length of 80 microglial processes and to delineate 20 somas from several ROIs in brain sections for each animal (control n=3; LPS-treated n=4). Statistical analysis was performed using unpaired t-test. Mean with SEM are shown. ****p <0.0001.

In the brain, while there was generally higher expression of TSPO protein in the LPS treated animals when compared to controls in the striatum (mean 2.334 vs 0.6130) and corpus callosum (mean 1.783 vs 0.54) regions, it did not achieve statistical significance possibly due to the limited sample size. However, we observed distinct changes in the morphological characteristics of the microglia such as the length of the glial processes and the size of the soma (**Figure 8**). The microglial processes were shorter and the cell bodies were larger in LPS treated rats (p<0.0001) indicating there was early microglial activation in these animals.

## Discussion

The sequential organ failure assessment (SOFA) or the shorter bedside clinical tool quickSOFA, have been implemented to evaluate groups of patients with SIRS/sepsis and better predict the severity of resulting organ dysfunction, morbidity, and mortality (42, 43). However, these tools are still limited in sensitivity and performance (44, 45), creating the need for more accurate measures of organ-level inflammation in septic patients. The effectiveness of [^18^F]DPA-714 as a biomarker of peripheral inflammation has previously been validated in different diseases (46–49). In this study, we used [^18^F]DPA-714 in a rat model of LPS-induced systemic inflammation, showing that *in vivo* whole-body PET imaging with the TSPO biomarker [^18^F]DPA-714 can be used to quantify organ-level immunoreactivity. As expected, [^18^F]DPA-714 PET imaging indicated increased expression of TSPO in the brain, lungs, bone marrow, and liver, and a variable change in TSPO expression in the spleen when compared to baseline (prior to LPS treatment) (**Figure 1**).

Sepsis is initially associated with an overwhelming release of cytokines and later on a phase of immune suppression with substantial immune cell depletion as a result of constant exposure to pro- and anti-inflammatory cytokines (50, 51). The production of IL-6, TNFα, IL-18, and IL-1 pro-inflammatory cytokines along with IL-10 (anti-inflammatory cytokine) is a hallmark response to sepsis. Our model of systemic sterile inflammatory shock follows this characteristic cytokine profile of sepsis showing increased expression of cytokines in the serum (**Figure 2**) and organ lysates (**Figure 3**). More importantly, we found a significant positive correlation between TSPO SUV_mean_ values in brain, lungs, bone marrow, and liver with the expression of these inflammatory cytokines in serum (**Figure 4**).

Our findings in the lungs are consistent with the current understanding of acute respiratory distress syndrome (ARDS) in severe SIRS and sepsis. In these patients, higher plasma levels of anti-inflammatory IL-10 during early course of disease correlate with severity of illness regardless of the use of oxygen support (52, 53) while higher expression of pro-inflammatory cytokines IL-1 and IL-6 at the onset of ARDS predicts unfavorable outcomes (54). After initial recovery from sepsis, continuous deployment of functionally impaired macrophages from lymphoid reservoirs to the lungs can further prevent lung recovery and increase negative outcomes during secondary infections (55, 56). In a previous study, [^18^F]FDG PET lung uptake was found to precede increased CT attenuation (lung edema) in a model of sepsis and ARDS, which was accompanied by neutrophil influx reflected by increased myeloperoxidase activity (57). In our model, we showed increased [^18^F]DPA-714 binding in the lungs of the LPS rats when compared to controls (**Figure 1C**) which correlated with systemic cytokines and was accompanied by increased staining for immune cell markers, namely macrophages, neutrophils, dendritic cells and lymphocytes (**Figure 6**), even though some of the differences in staining did not reach statistical significance. The latter however could be attributed to the small sample number used for IHC staining. As expected, TSPO staining colocalized mainly with macrophages (CD68), and to a lesser extent with other activated myeloid and lymphoid immune cells (**Supplementary Figures 2**, **3**). Our imaging findings thus support an inflammatory reaction induced in the lungs through the systemic administration of LPS, similar to what occurs in sepsis, that is measurable using [^18^F]DPA-714.

SIRS and sepsis can also affect the cell components of the bone marrow in the early stages, making it susceptible to inflammation, and showing increased proliferating cells in response to peripheral immune cell depletion (58). Using a repeated measures correlation to analyze bone marrow SUV_mean_ with serum cytokines, we showed a positive correlation with IL-6, IL-10, IL-1β, and VEGF, among others (**Figure 4**). The correlation between SUV and VEGF is most relevant as increased expression of this cytokine mediates morbidity and mortality in patients with severe sepsis. IL-18 shares similar characteristics with IL-1β and is increased in septic patients, particularly in those with thrombocytopenia (59, 60). In addition, we found negative correlations between bone marrow SUV_mean_ values and white blood counts (WBC (K/uL r= -0.86, p= 0.003), platelets (K/uL; r= -0.97, p= 0.00001) and lymphocytes (K/uL; r= -0.87, p= 0.002). Increased TSPO expression in the bone marrow is thus likely to reflect a combination of inflammation and increased hematopoiesis in response to peripheral leukopenia and thrombocytopenia, which were seen in our animal model and are commonly encountered in septic patients (**Supplementary Figure 1**).

Another peripheral organ evaluated in this study was the liver where we found an agreement between the PET imaging results and TSPO gene expression changes (**Figures 1**, **5**). Similarly, the imaging data correlated with increased Leptin and MIP-2 in serum which is an important finding since increased levels of Leptin and MIP-2 in septic patients also correlate with disease severity (61, 62). We did not assess liver enzyme levels in our study due to logistical limitations, however liver dysfunction is a well-known complication of sepsis. The association between the degree of liver inflammation and acute/chronic dysfunction is thus an important potential use for our quantitative noninvasive *in vivo* imaging approach.

When assessing the spleen, our PET data showed generally mixed change in binding of [^18^F]DPA-714 in the LPS group, with most animals showing decreased binding and some animals showing increased binding (**Figure 1**). No significant difference in TSPO gene expression (**Figure 5C**) were observed in the spleens of the LPS injected rats when compared to controls. Similar to PET imaging, IHC showed a mixed picture where two out of four animals showed increased TSPO staining. In one animal with increased TSPO staining despite decreased CD68 staining (**Figure 7**) we found increased B220^+^ cells and mixed changes of granulocytes and CD3^+^ cells. Increased TSPO staining in this case could be explained by lymphocytic proliferation despite migration of monocytes to other organs such as the lungs.

[^18^F]DPA-714 PET imaging of TSPO in the brain has been very well characterized in different models of neuroinflammation (17, 63–72). Our study shows a significant positive correlation between [^18^F]DPA-714 brain binding and levels of MCP-1, IL-2, EGF, IL-17A, and TNFα in serum. These cytokines are neuroregulatory molecules that penetrate the blood brain barrier and regulate interactions between peripheral tissues and the CNS (73, 74). They promote neutrophil mobilization to sites of inflammation and are increased in septic encephalopathy (75–77). Furthermore, IL-17A has been described as a main player in the immunological dysfunction during sepsis, with increased levels in serum of pediatric and adult patients during early stage of sepsis, making it an attractive biomarker and therapeutic target (78). When staining microglia with Iba1, we did not find differences in staining intensity between controls and LPS animals. We are aware that, despite being commonly used as a marker of microglial activation, Iba1 protein is not always specific enough to discriminate between activated and non-activated microglia, or to distinguish microglia from macrophages. Instead, we relied on the detection of subtle morphological changes in Iba1 stained microglia which are generally believed to specifically reflect stages of microglial activation. Our LPS animal model shows the morphological transformation of resting microglia into cells with less complex ramifications, thickening of the cell body, and presentation of an amoebic phenotype which are characteristic of microglial activation and phagocytic stage (**Figure 8**). This is similar to findings of microglial activation in the white matter of patients with systemic sepsis (enlarged and amoebic microglial phenotypes) when compared to non-septic controls (79). Sepsis survivors also can show clinical manifestations of delirium or long-term cognitive decline (5, 80), and other studies correlate these manifestations with white matter disruption (81). Neuroinflammatory changes in association with LPS administration and increased cytokine levels in the serum in our model could thus simulate brain-specific inflammatory changes in the setting of SIRS and sepsis. Whether those changes are associated with permanent damage, however, remains unclear and requires further evaluation.

In three animals who were imaged with [^18^F] DPA-714, we noted that responses were different from the rest of the cohort: one animal showed decreased TSPO binding both in the brain and lungs after LPS administration compared to baseline, and manifested only mild immune activation based on serum cytokines, one animal had mildly decreased binding in the brain and one animal showed lower binding in the lungs. The immune response of those last two animals, however, were within the range or on the higher end of cytokine expression levels. We thus believe these findings are due to natural variability in the immune response to LPS as well as variability in organ response to inflammatory signals, a phenomenon that has been previously described (82–84). By using a relatively larger sample number, we have confidence we have encompassed the whole spectrum of immune activation.

It is important to clarify here that although LPS can induce a systemic sterile inflammatory shock that can be similar in some aspects to what happens in SIRS and sepsis, direct translation of rodent findings into human findings in the setting of bacterial sepsis is not straightforward. LPS is an endotoxin present in the outer membrane of Gram-negative bacteria and extracted LPS has been broadly used in animal models and human studies to mimic the systemic inflammation caused by bacterial infections. There are however still differences in innate and adaptive responses between LPS and bacterial induced shock, including variability in serum cytokines and primary type of circulating leukocytes (85). To this end, however, our study is meant as a proof of concept that TSPO imaging can be useful in assessing organ-level inflammation, irrespective of the type of systemic inflammation.

One limitation of this study is that collection of tissues at baseline and after LPS administration for further biological analyses from the same animal is not possible. This forced us to use another set of animals for additional biological studies to support the PET data. Also, the small sample size (controls=3, LPS=4) used for immunohistochemistry could have limited the statistical analysis. However, with the promising results presented herein, additional studies with a larger sample size are warranted. Although [^18^F]DPA-714 has shown better affinity and specificity to TSPO than previous ligands, assessment of TSPO gene polymorphisms and binding status (86) should be performed in human studies for accurate quantitative analysis. This will allow further evaluation of correlations between [^18^F]DPA-714 uptake (or other TSPO ligands) and other molecular and clinical scores.

In conclusion, while the use of blood biomarkers and scoring systems can help predict organ dysfunction and mortality in patients with sepsis (87, 88), our study demonstrated that immunoreactivity in different organs (lung, liver, bone marrow, brain, and spleen) can be measured *in vivo* using the PET radiotracer [^18^F]DPA-714. Inflammatory changes in the lungs, liver, brain, and bone marrow correlated with peripheral inflammation (increased pro- and anti-inflammatory cytokines in serum such as TNFα, IL-1, IL-6, IL-17A, IL-18, and IL-10). Our study is a proof of concept of the feasibility of using PET to assess organ level immunoreactivity in systemic sterile inflammatory response and the same approach could potentially be used in sepsis to evaluate the effectiveness of preventative and therapeutic approaches in decreasing/controlling organ-level inflammation.

## Data availability statement

The original contributions presented in the study are included in the article/**Supplementary Material**. Further inquiries can be directed to the corresponding author.

## Ethics statement

The animal study was reviewed and approved by the Animal Care and Use Committee (ACUC) of the Clinical Center (CC) at the (NIH) and were performed in an AAALAC International accredited facility in accordance with relevant NIH policies and the Animal Welfare Act and Regulations.

## Author contributions

There are eleven co-authors who have contributed significantly to this paper: DH, SwS, WR and SaS conceived of and designed the study. WR, SwS, SaS, WS-S, NM-O, JL and ZW evaluated the animals, performed the scans, and collected/or analyzed the data. NM-O, ST and SwS performed *in vitro* experiments and analysis. FB synthesized and validated the radioactive compounds. DM performed IHC experiments and associated data preparation. ST, NM-O, SwS and DH wrote the first draft of the paper. All authors participated in drafting the article and/or revising it critically for intellectual content. All authors contributed to the article and approved the submitted version.

## Funding

Funding for this study was provided by the Intramural Research Program of the Clinical Center, NIH (Center for Infectious Disease Imaging (CIDI), Radiology and Imaging Sciences).

## Conflict of interest

The authors declare that the research was conducted in the absence of any commercial or financial relationships that could be construed as a potential conflict of interest.

## Publisher’s note

All claims expressed in this article are solely those of the authors and do not necessarily represent those of their affiliated organizations, or those of the publisher, the editors and the reviewers. Any product that may be evaluated in this article, or claim that may be made by its manufacturer, is not guaranteed or endorsed by the publisher.

## Supplementary material

Supplementary Data Sheet 2 Graphical abstract.
