# Supplementary material for: Assessing organ-level immunoreactivity in a rat model of sepsis using TSPO PET imaging
Source: Front Immunol. 2022 Nov 10;13:1010263. doi: 10.3389/fimmu.2022.1010263 (PMC9685400; doi:10.3389/fimmu.2022.1010263)
Supplement: Supplementary file 1 [file DataSheet_1.pdf]

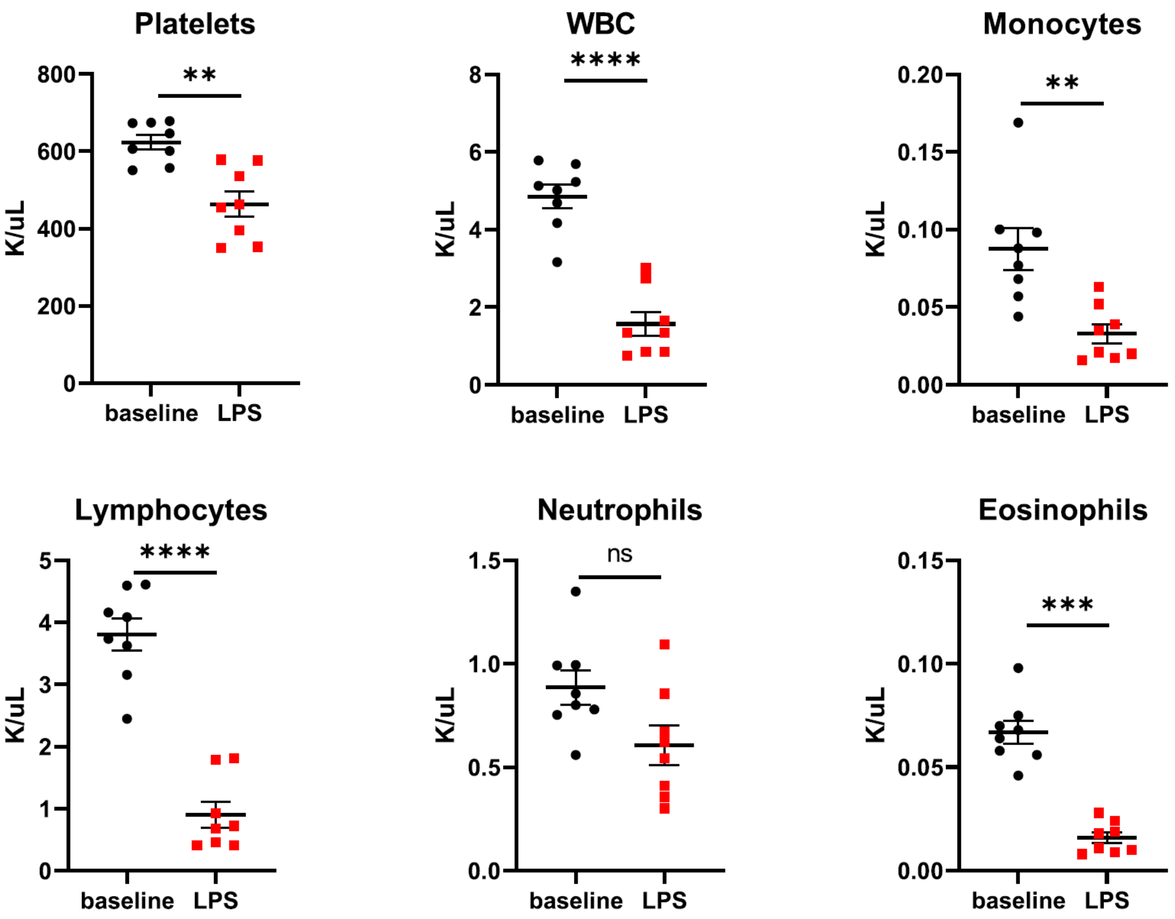

Supplementary Figure 1: Complete blood count

Post-LPS treated rats show changes in blood cell populations which is characterized by depletion of platelets ($p=0.0069$), WBC ($p<0.0001$), monocytes ($p=0.0088$), lymphocytes ($p<0.0001$), neutrophils ($p=0.0526$), and eosinophils ($p=0.0003$) when compared to baseline ($n=8$). Statistical analysis performed was paired t-test. Mean with SEM are shown. p-values <0.05 are considered statistically significant.

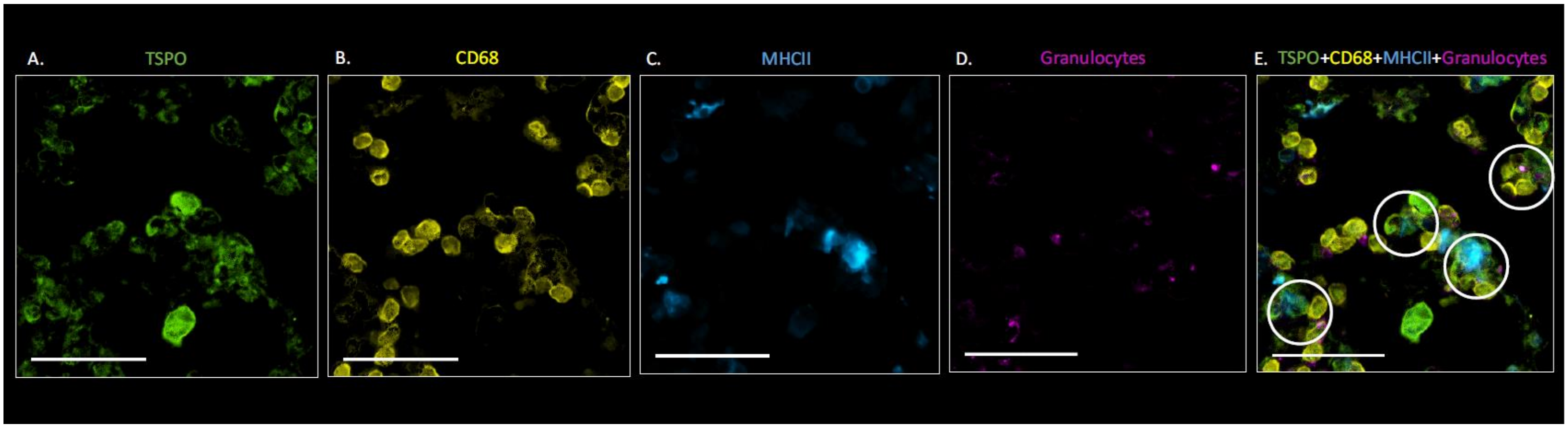

Supplementary Figure 2: Co-staining of TSPO with myeloid cells in lung sections of LPS treated rat

Representative MF-IHC image of a lung section from an LPS treated rat showing staining with (A) TSPO, (B) CD68 (macrophages), (C) MHCII (dendritic cells), (D) granulocytes (neutrophils) and the (E) merged image with all four stains. The white circles in the merged image indicate co-localization of macrophages, dendritic cells, or neutrophils with TSPO.

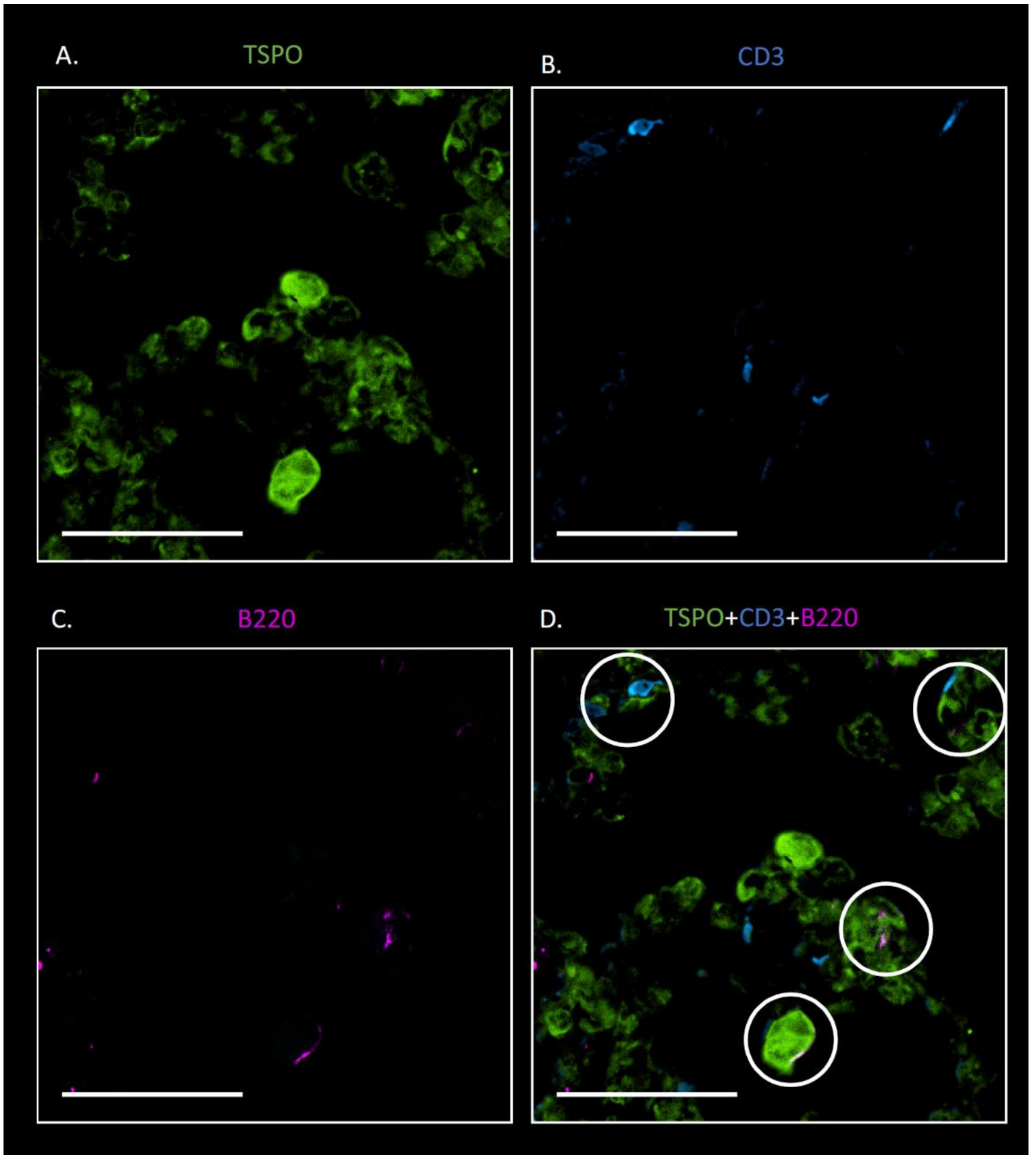

Supplementary Figure 3: Co-staining of TSPO with lymphoid cells in lung sections of LPS treated rat

Representative MF-IHC image of a lung section from an LPS treated rat showing staining with (A) TSPO, (B) CD3 (T cells), (C) B220 (B cells) and the (D) merged image with all three stains. The white circles in the merged image indicate co-localization of T cells or B cells with TSPO.
