# Supplementary material for: Assessing organ-level immunoreactivity in a rat model of sepsis using TSPO PET imaging
Source: Front Immunol. 2022 Nov 10;13:1010263. doi: 10.3389/fimmu.2022.1010263 (PMC9685400; doi:10.3389/fimmu.2022.1010263)
Supplement: Supplementary Data Sheet 2 — Graphical abstract. [file DataSheet_2.pdf]

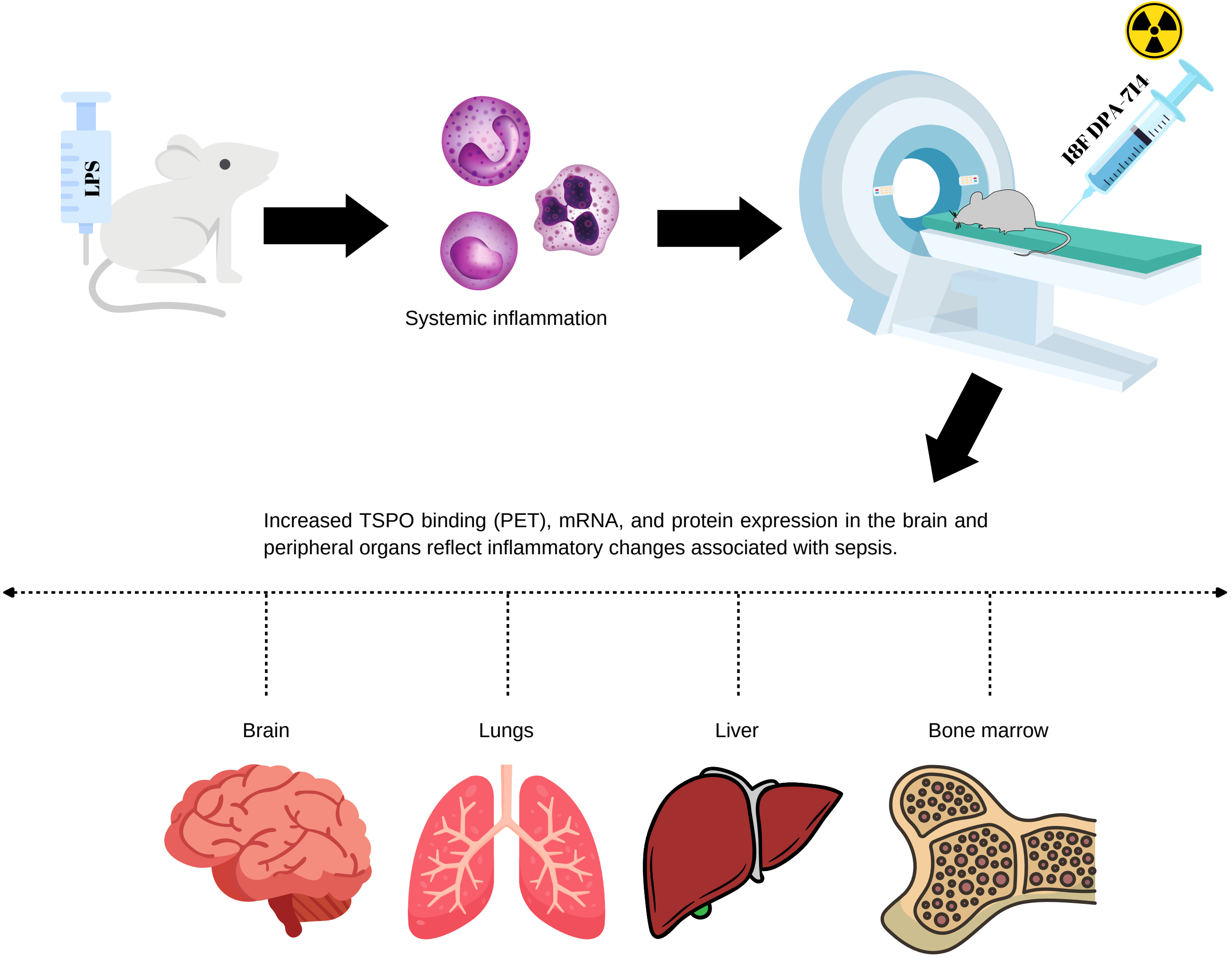
LPS
Systemic inflammation
18F DPA-714
Increased TSPO binding (PET), mRNA, and protein expression in the brain and peripheral organs reflect inflammatory changes associated with sepsis.
Brain
Lungs
Liver
Bone marrow
